# Decrease of miR-146b-5p in Monocytes during Obesity Is Associated with Loss of the Anti-Inflammatory but Not Insulin Signaling Action of Adiponectin

**DOI:** 10.1371/journal.pone.0032794

**Published:** 2012-02-29

**Authors:** Maarten Hulsmans, Els Van Dooren, Chantal Mathieu, Paul Holvoet

**Affiliations:** 1 Atherosclerosis and Metabolism Unit, Department of Cardiovascular Sciences, KU Leuven, Leuven, Belgium; 2 Clinical and Experimental Endocrinology Unit, Department of Clinical and Experimental Medicine, KU Leuven, Leuven, Belgium; National Institutes of Health, United States of America

## Abstract

**Background:**

Low adiponectin, a well-recognized antidiabetic adipokine, has been associated with obesity-related inflammation, oxidative stress and insulin resistance. Globular adiponectin is an important regulator of the interleukin-1 receptor-associated kinase (IRAK)/NFκB pathway in monocytes of obese subjects. It protects against inflammation and oxidative stress by inducing IRAK3. microRNA (miR)-146b-5p inhibits NFκB-mediated inflammation by targeted repression of IRAK1 and TNF receptor-associated factor-6 (TRAF6). Therefore, we measured the expression of miR-146b-5p in monocytes of obese subjects. Because it was low we determined the involvement of this miR in the anti-inflammatory, antioxidative and insulin signaling action of globular adiponectin.

**Methods:**

miR-146b-5p expression in monocytes of obese subjects was determined by qRT-PCR. The effect of miR-146b-5p silencing on molecular markers of inflammation, oxidative stress and insulin signaling and the association with globular adiponectin was assessed in human THP-1 monocytes.

**Results:**

miR-146b-5p was downregulated in monocytes of obese persons. Low globular adiponectin decreased miR-146b-5p and IRAK3 in THP-1 monocytes, associated with increased mitochondrial reactive oxygen species (ROS). Intracellular ROS and insulin receptor substrate-1 (IRS1) protein were unchanged. Silencing of miR-146b-5p with an antisense inhibitor resulted in increased expression of IRAK1 and TRAF6 leading to more NFκB p65 DNA binding activity and *TNFα*. As a response IRAK3 and IRS1 protein increased. Mitochondrial and intracellular ROS production did not increase despite more inflammation. In addition, exposure of miR-146b-5p-depleted THP-1 monocytes to high levels of globular adiponectin resulted in an increased production of TNFα and intracellular ROS. Still, they did not lose their potential to increase IRAK3 and IRS1 protein and to decrease mitochondrial ROS.

**Conclusion:**

miR-146b-5p, decreased in monocytes during obesity, is a major mediator of the anti-inflammatory action of globular adiponectin. It appears not to be involved in insulin signaling possibly by protective response of IRAK3 and lack of mitochondrial ROS production.

## Introduction

Over the past decade, it has become quite clear that chronic, low-grade inflammation and oxidative stress play a key role in the initiation, propagation and development of obesity and associated metabolic disorders like insulin resistance, type 2 diabetes, metabolic syndrome and cardiovascular disease [1]–[5]. Maladaptive production of various adipokines (e.g. adiponectin, resistin, visfatin, and leptin), and monocyte migration and subsequent transformation into macrophages within diseased tissues are key factors in the self-perpetuating inflammation associated with metabolic disorders [6], [7]. In particular, plasma levels of adiponectin are significantly lower in obese individuals and have been associated with inflammation, insulin resistance and the development of cardiovascular disease. Adiponectin is present in the plasma in its full length, forming homomultimers, and as globular adiponectin, a shorter product formed by macrophage-dependent elastase cleavage [8]. Globular adiponectin appears to be responsible for most biological effects of adiponectin [9], [10]. The protective effect of adiponectin has been attributed to its anti-inflammatory action [11].

The increased adipose tissue macrophage populations during obesity require an influx of circulating monocytes. Indeed, obesity is associated with increased levels of blood monocytes [12], [13], which are already activated in the circulation and are characterized by an increase in NFκB activity, an increase in the transcription of pro-inflammatory genes (e.g. tumor necrosis factor-α (TNFα)) and an increase in the production of reactive oxygen species (ROS) [14]. Our lab has previously shown that obesity-associated low levels of globular adiponectin decreases the expression of interleukin-1 receptor-associated kinase-3 (IRAK3) in monocytes [15]. IRAK3 is exclusively expressed in monocytes/macrophages, acts as an inhibitor of the IRAK/NFκB-mediated inflammation and is necessary for the anti-inflammatory and antioxidative action of globular adiponectin. Low levels of IRAK3 in monocytes are associated with increased production of *TNFα* and ROS despite the increase in mitochondrial antioxidant *superoxide dismutase-2* (*SOD2*) [15]. The increased production of mitochondrial ROS (mROS) in monocytes induces insulin resistance at the molecular level facilitating the development of diabetes and atherosclerosis [16]–[19].

microRNAs (miRs) are a class of small endogenous non-coding RNAs (∼22 nt), which function as important regulators of a wide range of cellular processes by modulating gene expression [20]. Two major silencing mechanisms have been identified for miRs: they can inhibit translational initiation or induce mRNA degradation [21]. Recent studies have shown that dysregulation of miR expression is closely associated with many diseases, including obesity, type 2 diabetes and atherosclerosis [22]–[25]. However, the possible role of miRs in the protective effect of adiponectin on monocytes remains to be determined. A possible candidate is miR-146b-5p. It has been shown that miR-146b-5p decreases the expression of TNFα in THP-1 monocytes by the targeted repression of IRAK1 and TNF receptor-associated factor-6 (TRAF6), two key adapter molecules in the IRAK/NFκB pathway [26], [27].

Here, we show that miR-146b-5p is decreased in circulating monocytes of obese subjects. Functionally, miR-146b-5p is regulated by variable globular adiponectin concentrations and acts as an inhibitor of NFκB-mediated inflammation. Furthermore, miR-146b-5p is necessary for the anti-inflammatory action of high levels of globular adiponectin. In contrast, miR-146b-5p appears not to be involved in the insulin signaling in monocytes possibly due to the protective response of IRAK3 and lack of mROS production.

## Results

### Characteristics of study cohort

The study cohort comprised 14 lean controls (29% male) and 21 morbidly obese individuals (33% male), without clinical symptoms of cardiovascular disease. Obese patients were more frequently diabetic and treated with a statin. They had higher IL-6, high sensitivity C-reactive protein (hs-CRP), leptin and glucose levels, and lower adiponectin levels, indicating chronic systemic inflammation. The higher levels of circulating oxidized LDL indicated systemic oxidative stress. Furthermore, insulin and triglyceride concentrations were higher; HDL-cholesterol was lower. Obese individuals had higher systolic and diastolic blood pressure. Insulin resistance, calculated by a homeostasis model assessment (HOMA-IR), was 86% higher in obese subjects (Table 1).

**Table 1:** Characteristics of obese subjects.

	Lean controls (n = 14)	Obese patients (n = 21)
Gender (% male)	29	33
Smoking (%)	7	19
T2DM (%)	0	38*
Statin use (%)	0	33*
Age (years)	33±3	39±3
BMI (kg/m^2^)	21±1	44±1***
Leptin (ng/ml)	8.7±1.4	65.6±8.0***
Adiponectin (µg/ml)	10.9±1.8	3.9±0.6**
Glucose (mg/dl)	83±2	111±7***
Insulin (mU/l)	10.3±1.8	16.5±2.1**
HOMA-IR	2.1±0.4	3.9±0.5**
Triglycerides (mg/dl)	80±7	132±11***
LDL-C (mg/dl)	110±9	85±6*
HDL-C (mg/dl)	64±4	49±3**
SBP (mmHg)	120±3	137±3**
DBP (mmHg)	75±3	86±2**
IL-6 (pg/ml)	1.8±0.2	4.8±0.4***
Hs-CRP (mg/l)	0.49±0.10	5.65±1.13***
Ox-LDL (IU/l)	50±5	71±4**

Data shown are means ± SEM.

* *P*<0.05,

** *P*<0.01 and,

*** *P*<0.001 obese compared with lean controls. Abbreviations: BMI, body mass index; C, cholesterol; DBP, diastolic blood pressure; HOMA-IR, homeostasis model assessment of insulin resistance; hs-CRP, high sensitivity C-reactive protein; ox-LDL, oxidized LDL; SBP, systolic blood pressure; T2DM, type 2 diabetes mellitus.

### miR-146b-5p is decreased in monocytes of obese subjects and is regulated by variable adiponectin concentrations

**Figure 1:**
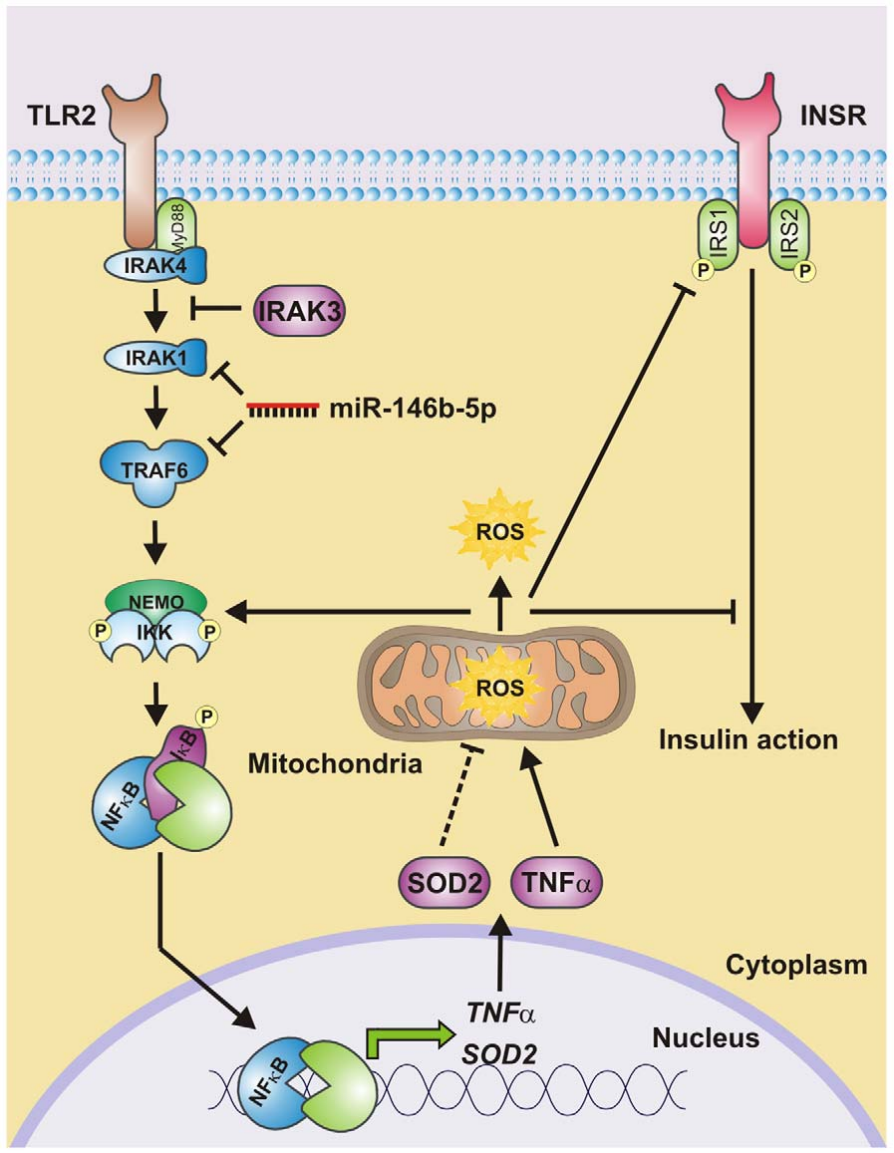
Theoretical model showing the interaction between miR-146b-5p, NFκB-mediated inflammation, ROS production and the insulin signaling. Activation of monocytes in the circulation is characterized by increased activation of the inflammatory toll-like receptor-2 (TLR2)/NFκB signaling pathway. Activation of this pathway will elicit pro-inflammatory cytokine release (e.g. tumor necrosis factor α (TNFα)), mitochondrial and intracellular ROS production and impairment of the insulin sensitivity at the molecular level. More mitochondrial ROS production is associated with an increased expression of the mitochondrial antioxidant *superoxide dismutase-2* (*SOD2*), suggesting that *SOD2* only acts as mitochondrial stress marker (indicated as dotted line). The formed ROS will not only induce the IKK complex phosphorylation and thereby the translocation of NFκB but will also lead to inhibition of the insulin receptor signaling pathway. Interestingly, interleukin-1 receptor-associated kinase-3 (IRAK3) and miR-146b-5p are located upstream of the transcription factor NFκB and negatively regulates TLR signaling. IRAK3 prevents the dissociation of IRAK1 and IRAK4 from MyD88 and formation of IRAK-TRAF6 complexes. miR-146b-5p, on the other hand, inhibits the translation of IRAK1 and TRAF6. Flow of the pathway at the protein interaction level is indicated by black arrows. Blunted arrows indicate inhibition. Phosphorylation is indicated by a circled P.

**Figure 2:**
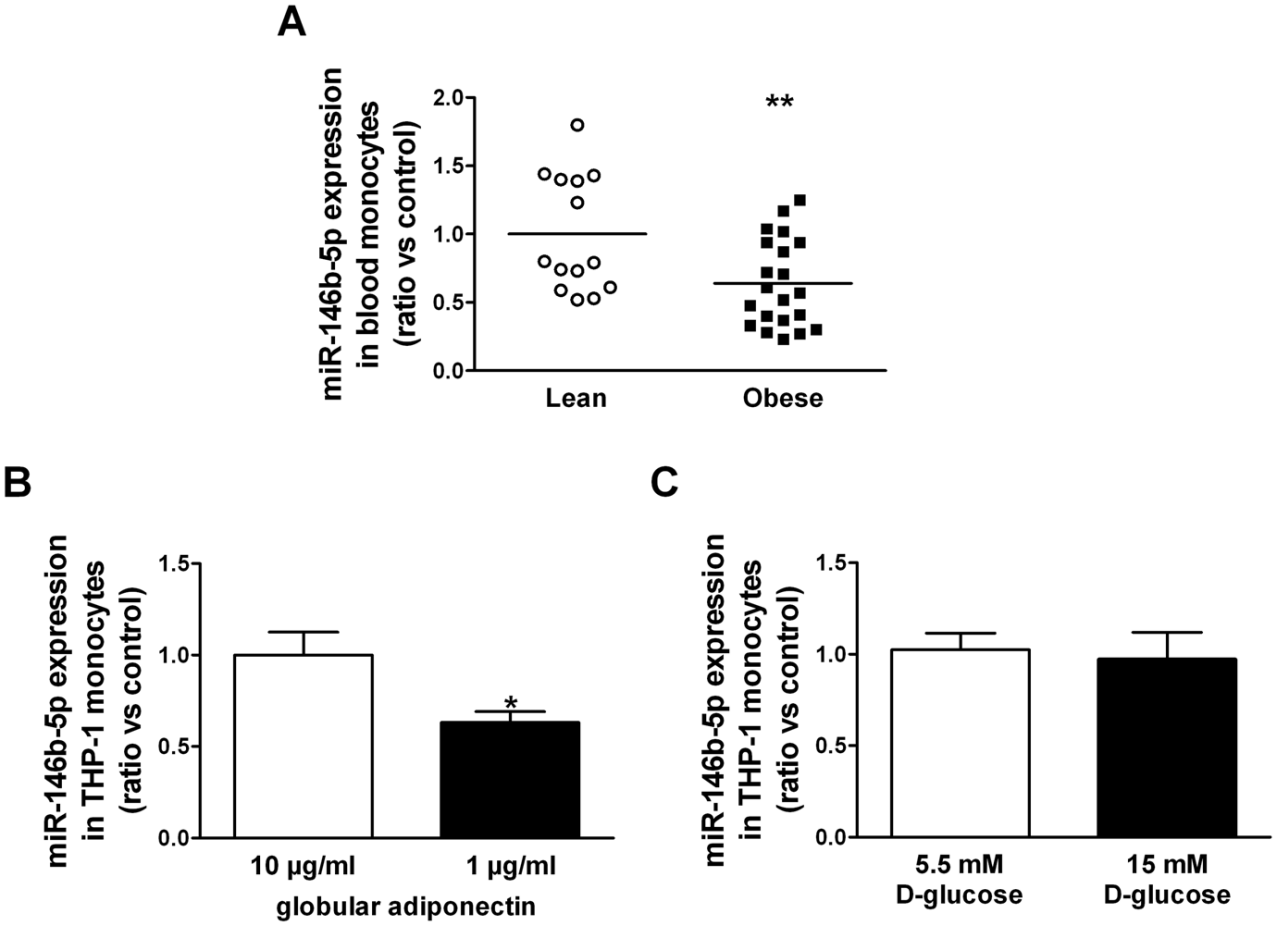
miR-146b-5p expression is decreased in monocytes of obese subjects and is regulated by globular adiponectin. (**A**) miR-146b-5p expression levels in isolated monocytes from 14 lean controls and 21 obese subjects as determined by qRT-PCR analysis. Data are expressed as means. ^**^ *P*<0.01 obese compared with lean controls. miR-146b-5p expression levels determined by qRT-PCR in THP-1 cells exposed to (**B**) 1 or 10 µg/ml globular adiponectin for 24 h (n = 6), and (**C**) 5.5 mM D-glucose and 9.5 mM D-mannitol (osmotic control) or 15 mM D-glucose for 24 h (n = 6). Data shown are means ± SEM. ^*^ *P*<0.05 compared with THP-1 cells exposed to 10 µg/ml globular adiponectin.

We determined the expression level of miR-146b-5p in isolated monocytes of 14 lean controls and 21 obese subjects. Figure 1 illustrates a theoretical model of the association between miR-146b-5p, NFκB-mediated inflammation, mROS and intracellular ROS (iROS) production and the insulin signaling by its predicted interaction with IRAK1 and TRAF6 in monocytes. The expression of the candidate miR was assessed by quantitative real-time PCR (qRT-PCR), and normalized by expression levels of *RNU5G*, identified as most stable reference gene (GeNorm [28]). miR-146b-5p was downregulated in monocytes of obese subjects (Figure 2A). The expression was not affected by gender differences. Gene expressions of key molecules in the gene cluster are depicted in Table 2.

**Table 2:** Gene expressions in monocytes of obese subjects.

	Lean controls (n = 14)	Obese patients (n = 21)
*INSR*	0.99±0.04	0.65±0.02***
*IRAK1*	0.99±0.04	1.21±0.04***
*IRAK3*	0.98±0.04	0.49±0.03***
*IRS1*	1.00±0.18	0.93±0.15
*IRS2*	1.05±0.16	1.01±0.06
*NEMO*	1.00±0.09	1.36±0.04***
*NFκB1*	1.01±0.05	0.92±0.03
*SOD2*	1.00±0.05	2.65±0.28***
*TLR2*	0.99±0.08	1.54±0.06***
*TNFα*	1.05±0.09	2.18±0.31***
*TRAF6*	0.98±0.32	2.26±0.25***

Data shown are means ± SEM.

*** *P*<0.001 obese compared with lean controls.

Previously, we showed that differential expressions of targets within the gene cluster depended on adiponectin and glucose levels [15]. Therefore, we determined miR-146b-5p expression levels in response to low and high levels of anti-inflammatory and antioxidative adiponectin and to ROS- and inflammation-inducing high levels of glucose. As previously shown, exposure of THP-1 cells to low levels of adiponectin resulted in a decreased expression of IRAK3 compared to cells exposed to high levels of adiponectin. This decrease was associated with more *TNFα* (+75%, *P*<0.001), *SOD2* (+51%, *P*<0.001) and mROS production (+20%, *P*<0.01). The iROS production and the expression of insulin receptor substrate-1 (IRS1) protein, a key player in the insulin signaling [29], were not affected. Exposure to low levels of adiponectin was associated with a decreased expression of miR-146b-5p (Figure 2B).

Short-term exposure of THP-1 monocytes to high glucose, comparable to those in obese patients, did not affect miR-146b-5p expression (Figure 2C).

### miR-146b-5p regulates inflammation but not oxidative stress and insulin signaling *in vitro*

**Figure 3:**
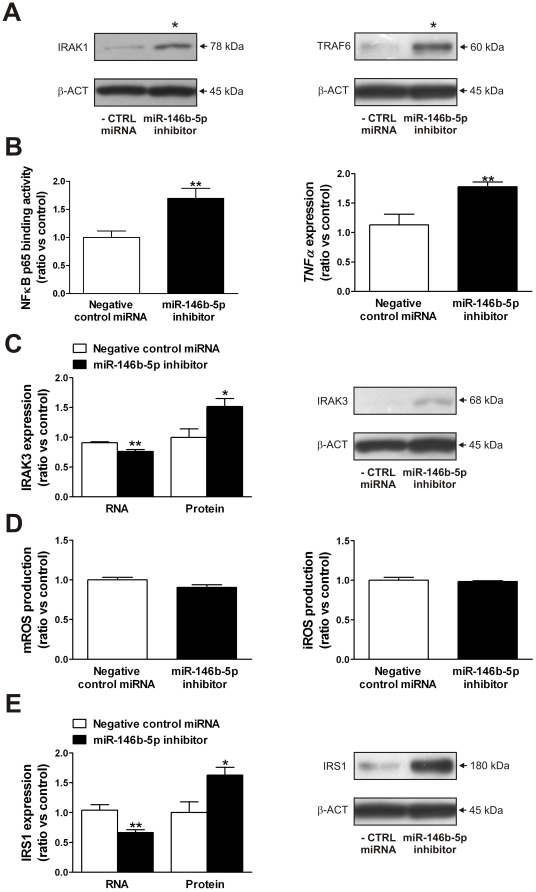
miR-146b-5p regulates inflammation in THP-1 monocytes. (**A**) IRAK1 and TRAF6 protein levels, (**B**) NFκB p65 DNA binding activity and *TNFα* RNA levels, (**C**) IRAK3 RNA and protein levels, (**D**) m- and iROS production and (**E**) IRS1 RNA and protein levels in THP-1 cells transfected with miR-146b-5p inhibitor as determined by qRT-PCR, ELISA, Western blotting and flow cytometry. Data are expressed as means ± SEM. n = 6. ^*^ *P*<0.05 and ^**^ *P*<0.01 compared with THP-1 cells transfected with negative control miRNA. Abbreviations: iROS, intracellular ROS; mROS, mitochondrial ROS; ROS, reactive oxygen species.

We transfected THP-1 monocytes with a LNA-modified miR inhibitor that targets miR-146b-5p. Depletion of miR-146b-5p in THP-1 monocytes increased IRAK1 and TRAF6 protein (Figure 3A) resulting in activation of the canonical NFκB signaling pathway, determined as NFκB p65 DNA binding activity, and an increased expression of *TNFα* (Figure 3B). The expression of IRAK3 was decreased on RNA level but increased on protein level (Figure 3C). We also determined the effect of miR-146b-5p knockdown on oxidative stress and insulin signaling by measuring ROS production and the expression levels of IRS1. The knockdown of miR-146b-5p in THP-1 monocytes had no effect on m- and iROS production (Figure 3D). The expression of *SOD2* was decreased (−36%, *P*<0.01) after miR-146b-5p depletion (data not shown). Furthermore, the expression of IRS1 was decreased on RNA level but increased on protein level (Figure 3E). The expression of two other mediators of insulin signaling, the *insulin receptor (INSR)* and *IRS2*, was unchanged (data not shown).

### miR-146b-5p is a mediator of the anti-inflammatory action of globular adiponectin

**Figure 4:**
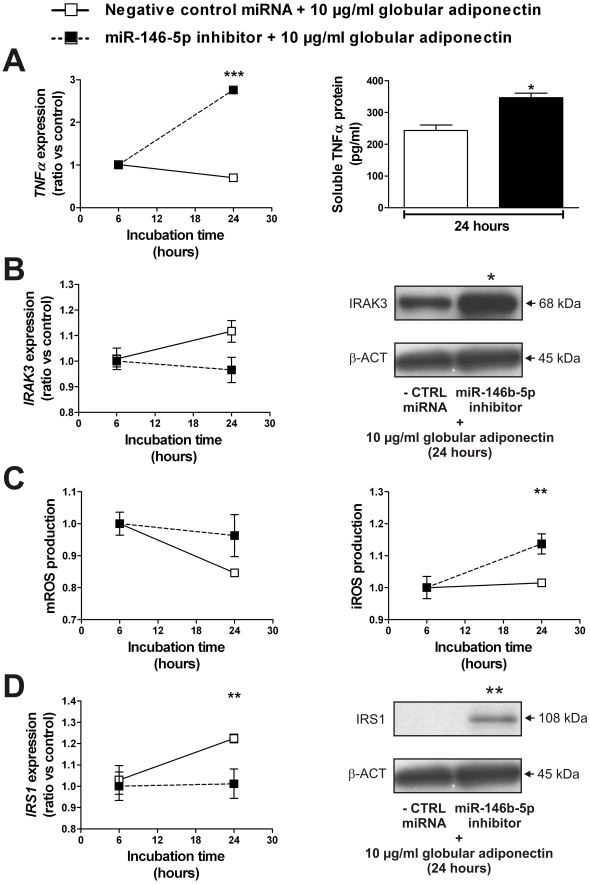
miR-146b-5p inhibition prevents the anti-inflammatory action of globular adiponectin. (**A**) TNFα RNA levels and soluble TNFα protein levels, (**B**) IRAK3 RNA and protein levels, (**C**) m- and iROS production, (**D**) IRS1 RNA and protein levels in THP-1 cells exposed to 10 µg/ml globular adiponectin with (n = 4) or without (n = 6) inhibition of miR-146b-5p. Data shown are means ± SEM. ^*^ *P*<0.05, ^**^ *P*<0.01 and ^***^ *P*<0.001 compared with THP-1 cells exposed to 10 µg/ml globular adiponectin without miR-146b-5p inhibition. Abbreviations: iROS, intracellular ROS; mROS, mitochondrial ROS; ROS, reactive oxygen species.

The association between miR-146b-5p and globular adiponectin (Figure 2B) suggests that miR-146b-5p is a possible mediator of the anti-inflammatory action of globular adiponectin. To study this interaction, we exposed miR-146b-5p-depleted THP-1 cells to 10 µg/ml globular adiponectin. The sequestration of miR-146b-5p and subsequent exposure to high levels of globular adiponectin resulted in an increased production of TNFα (RNA and soluble protein), IRAK3 protein and iROS (Figure 4A–C). The mROS production was not affected (Figure 4C). The IRS1 expression was decreased on RNA level but increased on protein level (Figure 4D). Furthermore, the *INSR* RNA expression was unchanged and *IRS2* RNA levels were increased (+16%, *P*<0.01).

## Discussion

Although recent evidence shows that adiponectin enhances insulin sensitivity and protects against chronic inflammation and oxidative stress in monocytes, the involvement of miRs in these protective effects of adiponectin has yet to be determined [15], [30]. We previously identified in monocytes of obese subjects IRAK3 as downregulated inhibitor of IRAK/NFκB-mediated chronic inflammation that is associated with oxidative stress and loss of protective properties of globular adiponectin [15]. In the present study, we identified miR-146b-5p as downregulated miR in monocytes of obese subjects with targets in the IRAK/NFκB-related gene cluster. We identified obesity-associated low levels of globular adiponectin as cause of the decrease in miR-146b-5p. In addition, miR-146b-5p is necessary for the anti-inflammatory action of globular adiponectin. According to the theoretical model depicted in Figure 1, we expected that silencing of miR-146b-5p would also lead to an increased ROS production and impaired insulin signaling. However, miR-146b-5p silencing did not have these effects most likely because in response to the downregulation of miR-146b-5p, IRAK3 was upregulated resulting in normalization of the mROS production and an increased IRS1 expression. The normalization of mROS was associated with a decrease in *SOD2* expression, which is in agreement with our previous data where we show that increased mROS is associated with more *SOD2* [15]. In aggregate, our current data together with our previous data [15] suggest that the combination of IRAK3 and miR-146b-5p is required for an optimal inhibition of TNFα-mediated inflammation, m- and iROS production and improved insulin signaling. In the absence of miR-146-5p, upregulation of IRAK3 can prevent mROS production associated with adequate IRS1 expression even in the presence of TNFα activation.

Mechanistically, miRs have been implicated as negative regulators of inflammatory processes at the post-transcriptional level [27]. Dysregulation of miR expression is associated with obesity and has profound effects on the onset and progression of associated metabolic disorders [25], [31]. miR-146b-5p is located on chromosome 10 and its expression is dependent on NFκB. It has been shown that miR-146b-5p decreases the expression of TNFα, IL-1β and IL-6 in THP-1 monocytes by the targeted repression of IRAK1 and TRAF6, two key adapter molecules in the IRAK/NFκB pathway [26], [27]. Moreover, miR-146b-5p is induced by the anti-inflammatory lipid mediator resolvin D1 in human macrophages implying that this miR is a key player in the resolution of inflammation by mediating the removal of components of the NFκB signaling pathway [32]. Furthermore, miR-146 is highly upregulated in monocytes during endotoxin tolerance and acts as a tuning mechanism to prevent an overstimulated inflammatory state [33]. The observed increase in NFκB p65 DNA binding activity and *TNFα* expression in miR-146b-5p-depleted THP-1 cells despite the increase in IRAK3 protein suggests that miR-146b-5p is a crucial negative regulator of the IRAK/NFκB pathway in monocytes. In contrast, the ROS production was unchanged after miR-146b-5p inhibition and the protein expression level of IRS1 was even increased. We hypothesize that this beneficial effect is due to the increase in IRAK3 protein and that the additional loss of IRAK3, as observed in obese patients, will increase ROS production and decrease insulin sensitivity in monocytes [15], [19].

The role of miR-146b-5p in protection against inflammation was further supported by the finding that after sequestration of miR-146b-5p the cells lost their potency to raise their anti-inflammatory action in response to high levels of adiponectin. The insulin sensitizing action of adiponectin was improved after knockdown of miR-146b-5p, possibly due to the increase in IRAK3. The loss of anti-inflammatory properties after miR-146b-5p depletion is also important in regard of adversative findings about the protective effects of adiponectin. Indeed, previous reports indicate that adiponectin suppresses inflammatory responses induced by hyperglycemia or TNFα [34], [35], while others have reported that adiponectin promotes inflammatory cytokine production and is associated with a significant increase in cardiovascular disease mortality in patients with metabolic diseases [36]–[39].

In summary, we have demonstrated a bidirectional relation between globular adiponectin and miR-146b-5p in monocytes: the expression level of miR-146b-5p is regulated by globular adiponectin and miR-146b-5p, at his turn, is necessary for the anti-inflammatory action of high levels of globular adiponectin. If deletion of miR-146b-5p leads to an increase of IRAK3, mROS production and insulin signaling appear to be controlled even in the presence of TNFα-mediated inflammation.

## Materials and Methods

### Materials

All chemicals were obtained from Sigma-Aldrich (Bornem, Belgium), unless stated otherwise. Monoclonal antibodies against β-ACT (13E5), IRAK1 (D51G7), IRS1 (D23G12) and TRAF6 (D21G3) were purchased from Cell Signaling Technology (Bioké, Leiden, Netherlands), and polyclonal anti-IRAK3 antibody from Rockland (Tebu-bio, Boechout, Belgium). Human THP-1 monocytic cells (TIB-202) were obtained from ATCC (LGC Standards, Molsheim, France).

### Patients and ethics statement

This study complies with the Declaration of Helsinki and the Medical Ethics Committee of the KU Leuven approved the study protocol. All human participants gave written informed consent. The patient cohort comprised 14 lean control (29% male; WCF<80 cm) and 21 obese individuals (33% male; WCF: 128±11 cm, mean ± SEM). The samples were collected at the Division of Endocrinology between March 29^th^, 2005 and May 30^th^, 2006. All participants were without symptoms of clinical atherosclerotic cardiovascular disease.

### Isolation of human monocytes

Blood samples were collected and after removal of the plasma fraction, peripheral blood mononuclear cells (PBMCs) were isolated using gradient separation on Histopaque-1077. Cells were washed three times in Ca^2+^- and Mg^2+^-free Dulbecco's (D)-PBS. PBMCs were incubated with CD14 microbeads (20 µl/1×10^7^ cells) for 15 min at 4°C. Cells were washed once and re-suspended in 500 µl Ca^2+^- and Mg^2+^-free DPBS containing 0.5% BSA/1×10^8^ cells. The suspension was then applied to an LS column in a MidiMACS Separator (Miltenyi, Leiden, Netherlands) [40], [41]. We selected CD14^+^ monocytes because CD14 intensity expression on circulating monocytes was found to be associated with increased inflammation in patients with diabetes [42].

### Blood analysis

Human blood samples were centrifuged to prepare plasma samples for analysis. Total and HDL-cholesterol and triglyceride levels were determined with enzymatic methods (Boehringer Ingelheim, Mannheim, Germany). LDL-cholesterol levels were calculated with the Friedewald formula. Insulin resistance was calculated by a homeostasis model assessment (HOMA-IR) = fasting plasma insulin (mU/l)×fasting blood glucose (mM)/22.5. Plasma glucose was measured with the glucose oxidase method (on Vitros 750XRC, Johnson & Johnson, New Brunswick, NJ, USA), and insulin with an immunoassay (Biosource Technologies, Invitrogen, Gent, Belgium). Ox-LDL [43] (Mercodia, Uppsala, Sweden), adiponectin, leptin and IL-6 were measured with ELISA (R&D Systems, Oxon, UK). Hs-CRP was measured on an Immage 800 Immunochemistry System (Beckman Coulter, Suarlée, Belgium). Blood pressure was taken three times with the participant in a seated position after 5 minutes quiet rest. The average of the last two measurements was used for systolic and diastolic blood pressure.

### RNA isolation and qRT-PCR analysis

Total RNA was extracted with TRIzol reagent (Invitrogen) and purified on miRNeasy Mini Kit columns (Qiagen, Venlo, Netherlands). The RNA quality was assessed with the RNA 6000 Nano assay kit using the Agilent 2100 Bioanalyzer; all samples achieved an RNA Integrity Number (RIN) score more than 8.0.

Total RNA (25 ng) was reverse transcribed in 20 µl reactions using the miRCURY LNA Universal RT miR cDNA synthesis kit (Exiqon, Vedbæk, Denmark), and the cDNA was diluted 80-fold. Each PCR was carried out in duplicate on a 7500 Fast Real-Time PCR system (Applied Biosystems, Gent, Belgium) in a total volume of 20 µl by using 8 µl of the diluted cDNA, 2 µl of LNA PCR primer set (hsa-miR-146b-5p: Exiqon, catalog no. 204553) and 10 µl of miRCURY LNA SYBRGreen master mix (Exiqon) according to the manufacturer's instructions. *RNU5G* was determined as most stable reference gene for each sample (GeNorm [28]). The relative gene expression was calculated by comparing cycle times for target PCR using the following equation: relative gene expression = 2^−(ΔCtsample—ΔCtcontrol)^.

For investigation of mRNA expressions, total RNA (0.5 µg) was reverse transcribed using SuperScript VILO cDNA synthesis kit (Invitrogen) as recommended by the manufacturer. The cDNA was diluted 50-fold. Primers used for qRT-PCR analysis are shown in Supplementary Table S1. PCR was performed with Fast SYBRGreen master mix (Applied Biosystems). Data were normalized to the housekeeping gene *β-ACT* as previously described [44].

### Cell culture

Human THP-1 monocytic cells were subcultured in RPMI 1640 (Gibco, Invitrogen) as described previously in detail [15], [44]. For globular adiponectin incubation experiments, cells were cultured at a density of 1×10^6^ cells/ml in RPMI 1640 supplemented with 10% FBS and 5 µg/ml gentamicin. After 24 h, 1 or 10 µg/ml globular adiponectin (PeproTech, London, UK) was added and the cells were incubated for 6 to 24 h. Globular adiponectin is a recombinant protein derived from human globular domain adiponectin cDNA expressed in *Escherichia coli*. This protein was endotoxin free (<2 EU/µg) according to the manufacturer. For glucose incubation experiments, cells were cultured at a density of 1×10^6^ cells/ml in glucose-free RPMI 1640 supplemented with 10% FBS, 5 µg/ml gentamicin, and 5.5 mM D-glucose in a 5% CO_2_ incubator at 37°C. After 24 h, 9.5 mM D-glucose or 9.5 mM D-mannitol (osmotic control) was added and incubated for 24 h under normal growth conditions. Cell viability, as determined by trypan blue exclusion, was >80%.

### Cell transfection

THP-1 cells were transiently transfected with a chemical synthesized mIRCURY LNA miR-146b-5p Power Inhibitor (5′-GCCTATGGAATTCAGTTCTC-3′, Exiqon). As negative control, we used miRCURY LNA miR Power Inhibitor Control (5′-GTGTAACACGTCTATACGCCCA-3′, Exiqon). Cells were transfected with 50 nM of miR inhibitor using HiPerfect reagent (Qiagen), according to the manufacturer's instructions with some modifications. Briefly, THP-1 cells were seeded at a density of 1.5×10^5^/well (24-well plate) in 100 µl of growth medium. Next, complexes (3 pmol of miR inhibitor plus 6 µl of HiPerfect reagent) were formed in 0.1 ml of serum-free RPMI-1640 for 10 min at room temperature and then added to each well. After 6 h of incubation under normal growth conditions, 400 µl of growth medium was added to each well and the cells were incubated for 42 h. miR-146b-5p-depleted THP-1 cells were exposed to globular adiponectin as mentioned above.

### Mitochondrial and intracellular ROS detection

To detect mitochondrial and intracellular ROS (mROS and iROS respectively) formation in treated THP-1 cells, measurements of MitoSOX Red and CellROX Deep Red (Invitrogen) fluorescence were performed by flow cytometry (Becton, Dickinson and Company, Aalst, Belgium). Cells were incubated with PBS containing 5 µM MitoSOX for 10 min or 2.5 µM CellROX for 30 min at 37°C and 5% CO_2_. The labeled cells were washed twice with PBS and then suspended in warm PBS for analysis by flow cytometry.

### NFκB p65 DNA binding activity

NFκB p65 DNA binding activity was assessed on isolated nuclear extracts of transfected THP-1 monocytes by ELISA using the TransAM NFκB p65 transcription factor assay kit according to the manufacturer's protocol (Active Motif, La Hulpe, Belgium). Briefly, 2 µg of nuclear extract diluted in complete lysis buffer was used in the p65 binding assay. The samples (in duplicate) were shaken for 1 h at room temperature in 30 µl binding buffer. After washing, anti-p65 antibody diluted 1∶1000 was applied to the wells for 1 h at room temperature. Specific binding was estimated by spectrophotometry after incubation with a horseradish peroxidase-conjugated antibody (1 h at room temperature, 1∶1000 diluted) at 450 nm wave length.

### Cytometric bead array (CBA) analysis of TNFα cytokine levels

Conditioned medium was harvested after centrifugation of treated THP-1 monocytes. Quantification of levels of soluble TNFα protein in the conditioned medium was performed using CBA Human TNFα Flex Set kit according to the manufacturer's instructions (Becton, Dickinson and Company). Briefly, undiluted medium was incubated with TNFα capture beads for 1 h at room temperature. Next, PE-conjugated detection antibody was added to the medium and the mixture was incubated for 1 h at room temperature. Finally, cytokine-bound beads were washed twice, and analyzed by flow cytometry.

### Western blotting

Western blot analysis was performed with 20 µg of total protein. Protein was electrophoresed through a 10–20% SDS-polyacrylamide gel (Bio-Rad, Nazareth Eke, Belgium) and transferred to a polyvinylidene difluoride membrane (Millipore, Brussel, Belgium). Membranes were processed according to standard Western blotting procedures. To detect protein levels, membranes were incubated with primary antibodies against β-ACT, IRAK1, IRAK3, IRS1 and TRAF6. The membranes were then incubated with horseradish peroxidase-conjugated secondary antibody (Santa Cruz Biotechnology, Tebu-bio, Boechout, Belgium) and developed with SuperSignal chemiluminescent substrate (Pierce, Thermo Fisher Scientific, Aalst, Belgium). A PC-based image analysis program was used to quantify the intensity of each band (Bio-1D, Vilber Lourmat, Marne-la-Vallée, France). Data were normalized to the housekeeping protein β-ACT.

### Statistical analysis

Lean and obese subjects were compared with an unpaired t-test (two-tailed); *in vitro* data were compared with the Mann–Whitney *U* test (GraphPad Prism 5, GraphPad Software, La Jolla, CA, USA). A *P*-value of less than 0.05 was considered statistically significant.

## Supporting Information

Table S1 Primers used in qRT-PCR.
(DOC)
