# Supplementary material for: Decrease of miR-146b-5p in Monocytes during Obesity Is Associated with Loss of the Anti-Inflammatory but Not Insulin Signaling Action of Adiponectin
Source: PLoS One. 2012 Feb 29;7(2):e32794. doi: 10.1371/journal.pone.0032794 (PMC3290617; doi:10.1371/journal.pone.0032794)
Supplement: Table S1 — Primers used in qRT-PCR. (DOC) [file pone.0032794.s001.doc]

**Table S1. Primers used in qRT-PCR.**

| **Gene** | **Forward primer** | **Reverse primer** |  |
| --- | --- | --- | --- |
| ***β-ACT*** | 5’-GGACCTGACCGACTACCTCATG-3’ | 5’-CGACGTAGCAGAGCTTCTCCTT-3’ |  |
| ***INSR*** | 5’-TGTGTACCTCTTGTGGCGTTTC-3’ | 5’-CTCAGTGCACCTCTCTCTTACATTG-3’ |  |
| ***IRAK1*** | 5’-TCAGTCCTAGCAAGAAGCGAGAA-3’ | 5’-ACTGGCCCGAGGTTGGA-3’ |  |
| ***IRAK3*** | 5’-TGCAACGCGGGCAAA-3’ | 5’-TTTAGTGATGTGGGAGGATCTTCA-3’ |  |
| ***IRS1*** | 5’-CATCCATTTCAGTTTGTTTACTTTATCC-3’ | 5’-TTATTCTGGTGTCACAGTGCATTTT-3’ |  |
| ***IRS2*** | 5’-GCTTCCCCAGTGCCTATCTTC-3’ | 5’-AAACCAACAACTTACATCTCCAATGA-3’ |  |
| ***NEMO*** | 5’-GCTGTCCCAAGTGCCAGTATC-3’ | 5’-CCGGCCCTACTCAATGCA-3’ |  |
| ***NFκB1*** | 5’-CCCTGACCTTGCCTATTTGC-3’ | 5’-CGGAAGAAAAGCTGTAAACATGAG-3’ |  |
| ***SOD2*** | 5’-TGGAAGCCATCAAACGTGACT-3’ | 5’-TTTGTAAGTGTCCCCGTTCCTT-3’ |  |
| ***TLR2*** | 5’-TGCAAGTACGAGCTGGACTTCTC-3’ | 5’-GTGTTCATTATCTTCCGCAGCTT-3’ |  |
| ***TRAF6*** | 5’-CATGAAAAGATGCAGAGGAATCAC-3’ | 5’-GAACAGCCTGGGCCAACAT-3’ |  |
| ***TNFα*** | 5’-CAAGCCTGTAGCCCATGTTGTA-3’ | 5’-TTGGCCAGGAGGGCATT-3’ |  |
